# ADI1, a methionine salvage pathway enzyme, is required for *Drosophila* fecundity

**DOI:** 10.1186/s12929-014-0064-4

**Published:** 2014-07-19

**Authors:** He-Yen Chou, Yu-Hung Lin, Guan-Lin Shiu, Hsiang-Yu Tang, Mei-Ling Cheng, Ming-Shi Shiao, Li-Mei Pai

**Affiliations:** 1 Graduate Institute of Biomedical Sciences, Chang Gung University, Tao-Yuan, Taiwan; 2 Department of Biochemistry, Chang Gung University, Tao-Yuan, Taiwan; 3 Chang Gung Molecular Medicine Research Center, Chang Gung University, Tao-Yuan, Taiwan; 4 Department of Biomedical Sciences, Chang Gung University, Tao-Yuan, Taiwan; 5 Healthy Aging Research Center, College of Medicine, Chang Gung University, Tao-Yuan, Taiwan

**Keywords:** ADI1, Methionine, MTA cycle, Fecundity, Drosophila

## Abstract

**Background:**

Methionine, an essential amino acid, is required for protein synthesis and normal cell metabolism. The transmethylation pathway and methionine salvage pathway (MTA cycle) are two major pathways regulating methionine metabolism. Recently, methionine has been reported to play a key role in *Drosophila* fecundity.

**Results:**

Here, we revealed that the MTA cycle plays a crucial role in *Drosophila* fecundity using the mutant of aci-reductone dioxygenase 1 (*D*ADI1), an enzyme in the MTA cycle. In dietary restriction condition, the egg production of *adi1* mutant flies was reduced compared to that of control flies. This fecundity defect in mutant flies was rescued by reintroduction of *D*adi1 gene. Moreover, a functional homolog of human ADI1 also recovered the reproduction defect, in which the enzymatic activity of human ADI1 is required for normal fecundity. Importantly, methionine supply rescued the fecundity defect in *Dadi1* mutant flies. The detailed analysis of *Dadi1* mutant ovaries revealed a dramatic change in the levels of methionine metabolism. In addition, we found that three compounds namely, methionine, SAM and Methionine sulfoxide, respectively, may be required for normal fecundity.

**Conclusions:**

In summary, these results suggest that ADI1, an MTA cycle enzyme, affects fly fecundity through the regulation of methionine metabolism.

## Background

Methionine, one of the essential amino acids, plays a key role in protein synthesis and cellular metabolic function, supplying sulfur and other compounds required for normal metabolism and cell growth [[1],[2]]. Methionine executes a well-known role for the initiation of protein synthesis in eukaryotes and prokaryotes. Indeed, the hydrophobic character of methionine is important for the binding of initiator tRNA to eIF-2, and most methionine residues are detected in the hydrophobic interior core of globular proteins [[3],[4]]. Previous studies have also shown that methionine is a source of the methyl groups that regulate the methylation of DNA and histones, and influence chromatin structure and gene expression in the liver [[5],[6]]. Methylation imbalance is correlated with several diseases including liver disease, cardiovascular disease and cancer [[7],[8]]. Methionine residues on the protein surface also function as endogenous antioxidants [[9],[10]]. Recent study also indicated that methionine plays a critical role in fecundity in *Drosophila* [[11]].

Except from diet, methionine contents are controlled by several pathways, including the folate pathway, transmethylation pathway and methionine salvage pathway. The folate pathway plays a pivotal role in one-carbon metabolism [[12],[13]]. In the transmethylation pathway, also called the methionine *de novo* pathway or methyl cycle, S-adenosylmethionine (SAM) is synthesized from ATP and methionine by SAM synthetase (SAM-S). The process of methyl cycle pathway is to metabolize SAM into S-adenosylhomocysteine (SAH) and subsequently to homocysteine. Then, homocysteine is converted into cysteine by trans-sulfuration or re-methylated to form methionine. SAM functions as a methyl donor involved in many biochemical reactions [[6],[14]]. The methionine salvage pathway, also termed the 5′-methylthioadenosine (MTA) cycle, is allowed to regenerate methionine from MTA and is also responsible for the production of polyamines which are critical for cell proliferation [[15],[16]]. The biochemical reactions in the MTA cycle are mainly carried out by six enzymes which are conserved from bacteria to yeast to human [[17],[18]]. However, the effects of the MTA cycle on methionine metabolism in *Drosophila* are still unknown.

In a previous study, Yeh et al. [[19]] identified Sip-L (hADI1), a hepatic factor capable of supporting HCV infection and replication in an otherwise non-permissive cell line. In a subsequent study, we demonstrated that human aci-reductone dioxygenase 1 (hADI1) over-expression in 293 cells enhances viral entry into cells but not replication of HCV [[20]]. ADI1, an MTA cycle enzyme, belongs to the cupin domain superfamily and has aci-reductone dioxygenase (ARD) enzymatic activity. The ADI1 associates with Fe^2+^ to produce formate and 2-keto-4-methylthiobutyrate (MTOB), the keto-acid precursor of methionine. Alternatively, ADI1 can associate with Ni^2+^ to produce formate, carbon monoxide and 3-methylthiopropionate [[21],[22]]. Previous studies of hADI1 have shown that it has multiple functions, such as the modulation of cell migration, apoptosis and RNA processing [[23]-[25]]. Despite these functions in basic cellular processes, the roles of ADI1 in whole animals are unknown. Therefore, we attempted to investigate the function of ADI1 in model animal *Drosophila*

In the present study, we generated the *Dadi1* null mutant and studied the role of *Dadi1* in fly fecundity. We found that the enzymatic activity of ADI1 is required for normal egg production, and human ADI1 is functionally exchangeable for this effect. From the metabolomic analysis, we concluded that three metabolites in methionine metabolism might be critical for *Drosophila* fecundity.

## Methods

### Fly strains

Deletions in *Dadi1* (*CG32068*) were generated by imprecise excision of P-element P{XP}CG32068^d01129^ (Exelixis Collection), which is located at 48 bp upstream of the start codon in exon 1 of the *Dadi1* gene. First, the P-element strain was crossed with the *rucuca* strain (Bloomington stock number 576), which carries third-chromosome lines marked with several recessive markers. The recombined P-element strain was mobilized using standard genetic methods by crossing to *Delta2-3/TM3,Sb* (carrying the transposase) and excision alleles were identified by loss of white marker. Three null mutants (*Dadi1*^*7*^, *Dadi1*^*9*^, *Dadi1*^*74*^) with deletions in the *Dadi1* gene and 11 hypomorphic alleles were selected from a collection of 508 excision strains by western blotting. Three null mutant alleles (*Dadi1*^*7*^, *Dadi1*^*9*^, *Dadi1*^*74*^) also verified the deletion region in the *Dadi1* gene by PCR amplification. The deletion region of *Dadi1*^*74*^ was from 10656504 to 10657550 in fly genomic DNA. The deletion region of *Dadi1*^*7*^ and *Dadi1*^*9*^ were 10656504–10658482 and 10655425–10657620, respectively. The lethality of *Dadi1* null mutants (*Dadi1*^*74*^ homozygote or *Dadi1*^*7*^*/Dadi1*^*9*^ trans-heterozygote) were shown to be less than 5%.

To remove most of the markers in *rucuca*, the *D*adi1^74^-FRT^80B^ strain was generated by crossing *D*adi1^74^ with P{neoFRT}80B/TM3Ser. The recombination was performed using standard genetics methods, and alleles were identified by containing two markers, *thread* (*th*) and *ebony (e)*. The *Dadi1*^74^-FRT^80B^ strain was used in this study for the fecundity assay. The P{neoFRT}80B strains were used in this paper for genetic background control.

To create the rescue construct P{UAST-*D*ADI1}, first, *Dadi1* cDNA encoding full-length *D*ADI1 was amplified by PCR, inserted with hemagglutinin (*HA*) epitope tag or without HA tag by *EcoRI-XhoI* sites, and introduced into the *pBluescript* (pBS) vector. Then, the *Eco*RI*-XhoI* fragment from pBS-HA-*D*ADI1 and pBS-*D*ADI1 were subcloned into the pUAST vector. The constructs were injected into *w minus* flies by *P*-element transformation. For the P{UAST-hADI1} and P{UAST-hADI1-E94A} constructs, full-length human AD1 cDNA was amplified from the human liver cDNA. The strategy used for the hADI1 constructs was the same as the one used for the P{UAST-*D*ADI1} constructs. For the P{UAST-hADI1-E94A} construct, site-direct mutagenesis was performed in the pBS-hADI1 construct. The pBS-hADI1-E94A was subcloned into pUAST and then injected into the *w minus* embryo.

### Western blotting

For western blot analysis, fly adult samples were dissected and homogenized gently in cell lysate buffer (50 mMTris-HCl, pH 7.5, 400mMNaCl, 5 mM EDTA, 1% Nonidet P-40 and protease inhibitor cocktail). Samples were left for 10 min on ice and then centrifuged at 14,000 rpm for 10 min at 4°C. The supernatant was then placed into a fresh centrifuge tube, protein sample buffer was added, and the sample was heated to 95°C for 10 min; this was followed by analysis by 12% SDS-PAGE. The proteins were then transferred to PVDF membrane and incubated for 1 hr in blocking buffer (7% nonfat milk in TBS/0.1% Tween-20). *D*ADI1, hADI1or alpha-tubulin (Sigma) antibody incubations were carried out first in blocking buffer for 16 hr at 4°C and then the membranes were washed with TBS/0.1% Tween-20. HRP-conjugated antibody was used as the secondary antibody for one hour. Finally, ECL substrate was added and protein signals were detected. For *D*ADI1 antibody generation, DNA fragment-encoding full-length *D*ADI1 was cleaved from pBS-*D*ADI1, inserted by *EcoRI-XhoI* sites, and introduced into the pQE81L (QIAGEN) vector. DH5α bacterial cells were transformed with pQE81L-*D*ADI1 and purified by Ni-NTA resin (QIAGEN). *D*ADI1 polyclonal antibody was raised in rabbits and rats by immunization with His-tag-*D*ADI1 fusion protein. The dilution of *D*ADI1 antibody used for western blotting was 1:500. For hADI1 antibody generation, full-length hADI1 was amplified by PCR, inserted by *EcoRI-XhoI* sites, and introduced into the pET32a vector. The His-tag-hADI1 fusion proteins were overexpressed in BL21 bacterial cells and purified by Ni-NTA resin. Human ADI1 polyclonal antibody was raised in rabbits and the dilution of hADI1 antibody was 1:500.

### Food conditions

For the fly food condition and fecundity assay, we used a protocol adapted from the one described in a study by Grandison et al. [[11]]. The restricted diet food contained 100 g BREWER’S Yeast, 50 g sucrose, 15 g agar, 3 ml propionic acid and 30 ml p-Hydroxy-benzoic acid methyl ester (3 g in 30 ml 95% ethanol) per liter. The fully-fed food was the same as the restricted diet food, except that the concentration of BREWER’S Yeast was increased to 200 g per liter. For methionine rescue experiments, the 0.7 or 1.4 mM methionine (Sigma, M9625) or 0.4 mM tryptophan (Sigma, T0254) was added to restricted diet food.

### Fecundity assay

Homozygous mutant flies (*Dadi1*^*74*^*/Dadi1*^*74*^) were generated by crossing male *Dadi1* heterozygous mutant flies with female *Dadi1* heterozygous mutant flies. Under the restricted diet condition, 135 *Dadi1* homozygous mutant female flies were mixed with 90 OreR male flies to mate for two days in 5 egg laying cups. The females were then separated from males and transferred to new egg laying cups with the restricted diet food. The density of flies was 30 females per egg laying cup. At least 90 females per one genotype were observed in various experiments. We regularly transferred flies to new egg laying cups after approximately 2 days and collected eggs on the appointed days. The eggs were counted on days 3, 6, 8, 11, 15, 22, 29 and 35, and a Student’s *t* test was used to investigate differences between the various genotypes.

### Targeted metabolites analysis

The methionine-associated metabolites were determined from wild-type and *Dadi1* homozygous mutant ovaries raised under the restricted diet condition. Twenty ovaries per genotype were collected and homogenized in 80% MeOH. The extraction samples were centrifuged at 12000 rpm for 10 min at 4°C. The supernatants were transferred into clean tubes, dried with N2, and stored at −80°C until UPLC/MS analysis. Samples were subjected to Ultra performance liquid chromatography coupled with triple quadrupole massspectrometry (UPLC/TQMS) system. Results were further analyzed using the Masslynx™ 4.0 and QuanLynx™ (Waters) software systems. The UPLC/TQMS analysis was conducted in the Metabolomics Core Laboratory of the Healthy Aging Research Center, Chang Gung University.

## Results

### The generation of *Drosophila adi1* mutants

**Figure 1:**
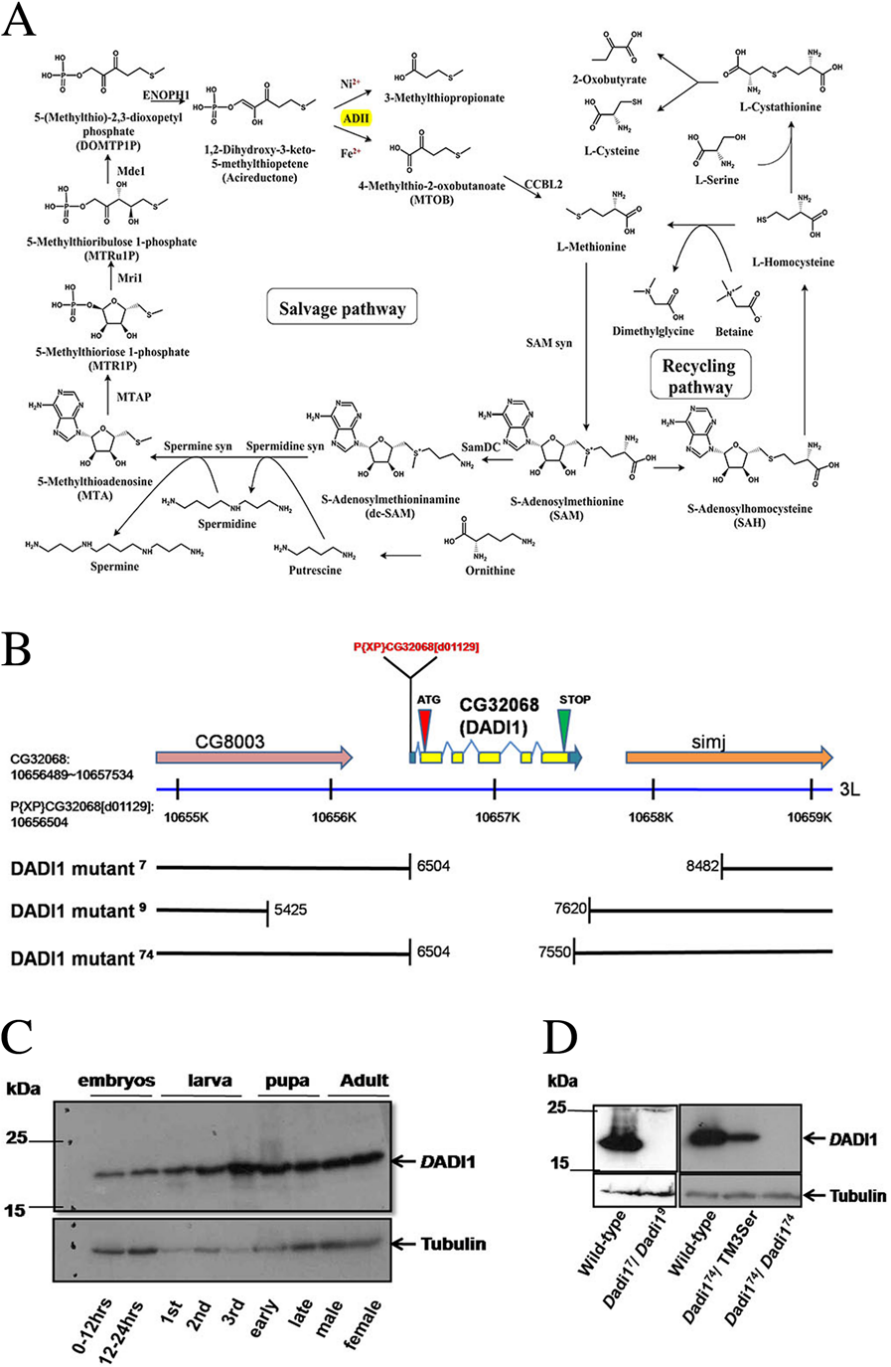
**Isolation of*D*adi1 alleles. (A)** Schematic diagram of the methionine salvage and recycling pathways, which is generated based on metabolism pathways in the Kyoto Encyclopedia Genes and Genomes (KEGG) database. The Main enzyme studied here is highlighted with yellow. **(B)** A map of genomic DNA displays the *Dadi1* (CG32068) locus. The *D*ADI1^d01129^ P-element insertion line (black arrow) contains a p-element at 48 bp upstream of the *D*ADI1 start codon (red arrow). Three *D*ADI1 mutant alleles (*Dadi1*^*7*^, *Dadi1*^*9*^ and *Dadi1*^*7*4^) were produced with alterations in the *Dadi1* coding region. **(C)** Immunoblotting of *D*ADI1 in several developmental stages. Embryos were collected after egg deposition. **(D)** The protein levels of *D*ADI1 were reduced in *Dadi1*^*74*^ heterozygote strain (*Dadi1*^*74*^/TM3Ser). Western blot analysis of *D*ADI1 protein levels of *Dadi1*^*7*^/ *Dadi1*^*9*^ trans-heterozygous alleles and *Dadi1*^*74*^ homozygous mutant indicated that they were protein null alleles.

To investigate the role of methionine metabolism in *Drosophila* fecundity, we generated *Dadi1* null mutants, by imprecise excision of the P element from strain P{XP}CG32068d01129 (Exelixis Collection). The *Drosophila adi1* gene encodes aci-reductone dioxygenase (ARD) and presumably functions in the MTA cycle (Figure 1A). It is located on chromosome 3 L at 67E6, and consists of five exons that encode a predicted protein of 186 amino acids (Flybase database ID CG32068). The P{XP}CG32068 d01129 P-element is located at 48 bp upstream of the start codon, in exon 1 of the *Dadi1* gene. Using genomic PCR amplification, three null mutants (*Dadi1*^*7*^, *Dadi1*^*9*^, *Dadi1*^*74*^) with deletions in the *Dadi1* gene were selected from a collection of 508 excision strains (Figure 1B). The deleted region of *Dadi1*^*74*^ includes an entire coding region of *Dadi1* gene but has no effects on neighboring genes, while the deleted regions of *D*adi1^7^ and *D*adi1^9^ are larger and include parts of the 5′ and 3′ neighboring genes, respectively. Therefore, homozygote *Dadi1*^*74*^ and trans-heterozygote of *Dadi1*^*7*^*/Dadi1*^*9*^ served as null mutants. More than 95% of the *Dadi1* null mutants eclosed to viable adults. Polyclonal anti-*D*ADI1 antibody raised against full-length protein, recognized one major band about 19 kDa in immunoblots of wild-type extracts from different developmental stages (Figure 1C). The ubiquitous expression of *D*ADI1 throughout all developmental stages suggested it could serve as an enzyme involved in metabolic reactions in fly life. A lack of proteins in null mutants was confirmed by a lack of signaling in western blotting for *Dadi1*^*7*^/*Dadi1*^*9*^ trans-heterozygous and *Dadi1*^*74*^ homozygous flies (Figure 1D). To our knowledge, the *Dadi1* mutant is the first mutant involved in the MTA cycle in *Drosophila*. Thus, the *Dadi1* mutant provides us with an opportunity to investigate the role of the MTA cycle in *Drosophila* development.

### *Dadi1* mutant females displayed a fecundity defect under dietary restriction

**Figure 2:**
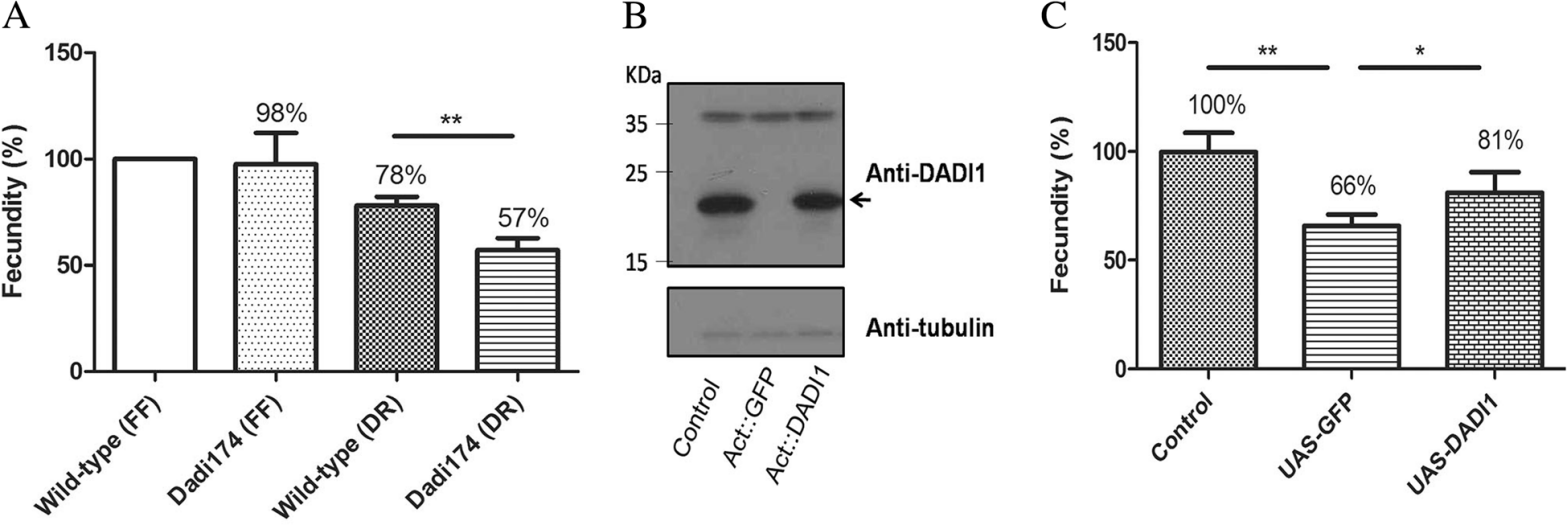
***D*ADI1 is required for*Drosophila*fecundity. (A)** Eggs of control and *Dadi1* mutant flies were enumerated and normalized to control flies under the dietary restriction condition. **(B)** Western blot analysis of *D*ADI1 protein levels in the adult lysate of control and *D*ADI1 transgenic lines, which expressed in *Dadi1* null mutant. **(C)** The fecundity defect was rescued in *Dadi1* mutant alleles expressing UAS-*D*ADI1 driven by Actin-Gal4. *P < 0.05, **P < 0.01.

MTA cycle enzymes are actively expressed in Drosophila ovaries (Flybase). Thus, we examined fecundity, the egg production ability, was examined in control and *Dadi1*^*74*^mutant female flies under different food conditions. Since protein supply is a major factor for growth and dietary restriction is known to reduce fecundity in flies [[11],[26],[27]], we designed both fully-fed and restricted diet conditions, which provided 20% and 10% yeast, respectively. Interestingly, under the restricted diet condition, *Dadi1*^*74*^ mutant females displayed a significant reduction in fecundity compared to that in the control females (Figure 2A), indicating that *Dadi1* is essential for fecundity when nutrition is poor. To verify whether this difference was caused by the lack of *Dadi1*, we expressed the exogenous *Dadi1* gene in the mutant flies. The ubiquitous expression of *D*ADI1 driven by actin-Gal4, indeed partially rescued the fecundity defect in *Dadi1* mutant females (Figure 2B-C).

### The enzymatic activity of *Dadi1* is required for the regulation of fly fecundity

**Figure 3:**
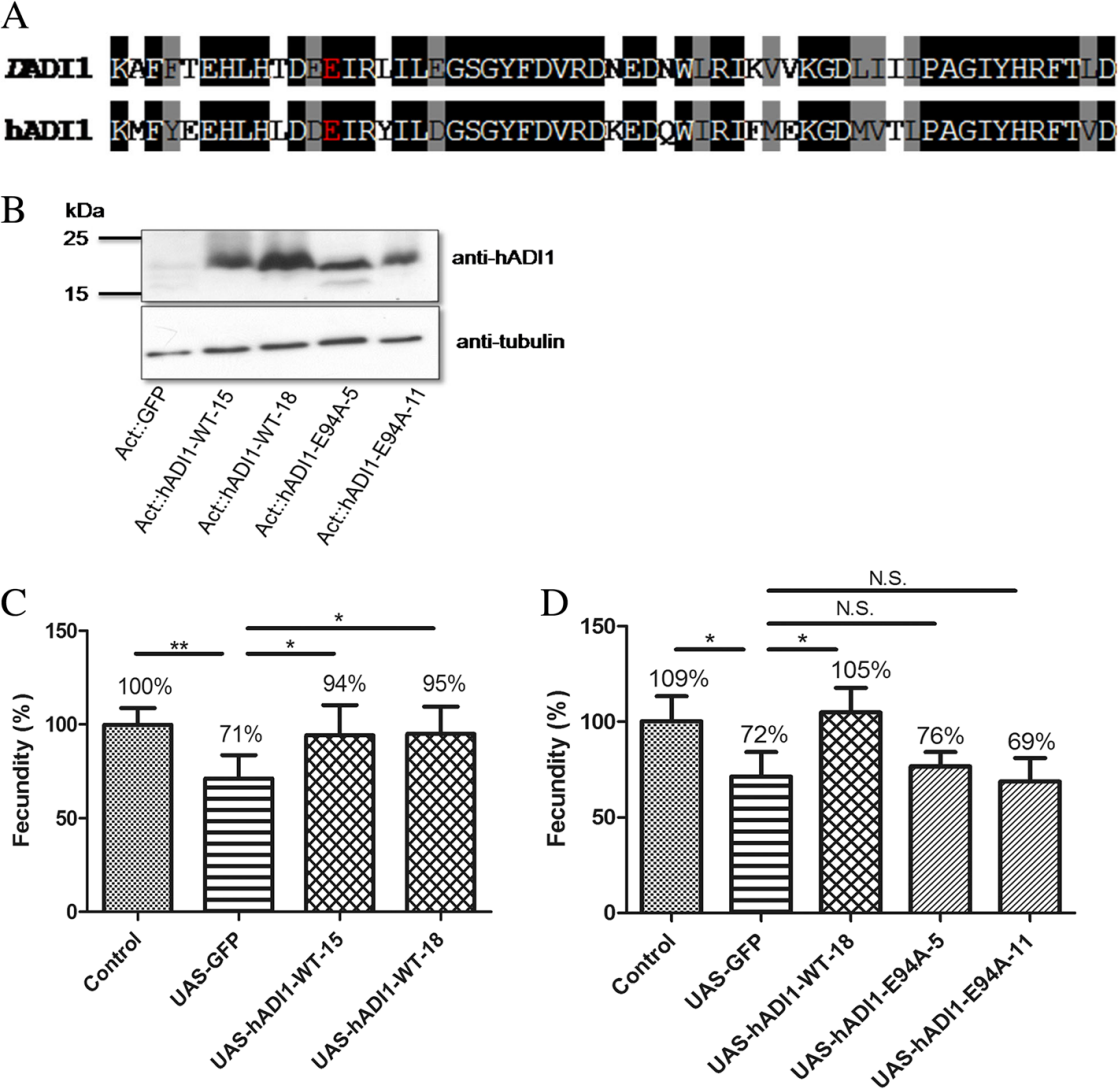
**Enzymatic activity of ADI1 is essential for regulating egg production. (A)** Protein sequence alignment is shown in the Cupin domain of hADI1 and *D*ADI1. The identity and similarity of Cupin domain is 68% and 84%, respectively. The glutamic acid site is required the ARD/ARD’ family to execute enzymatic function (red star). **(B)** The protein expression levels of human wild-type and E94A mutant transgenic lines were observed in the *Dadi1* null mutant background by hADI1 antibody. **(C)** Expressing human ADI1 by Actin-Gal4 can rescue the egg production defect in *Dadi1* mutant alleles. **(D)** The fecundity was not rescued by diverse expressing enzyme dead mutant (E94A) transgenic lines. *P < 0.05, **P < 0.01 and N.S., no significance.

ADI1 family proteins are highly conserved among different species from bacteria to human [[23]], and hADI1 shared 50% identity and 69% similarity with *D*ADI1 (CG32068) in protein sequence alignment (by NCBI-blast). The enzyme activity of this protein family is located in the highly conserved ARD domain, and *D*ADI1 showed 68% identity and 84% similarity to human ADI1 on this domain (Figure 3A). Previous studies have reported that human ADI1 obtains the ARD activity and functionally replaces yeast ADI1 in the in vivo enzyme activity assay [[21]]. Furthermore, a critical glutamic acid at the 94th residue has been demonstrated to be essential for its enzyme activity [[22],[25]]. In this study, we generated transgenic flies expressing a wild type hADI1or a hADI1-E94A mutant in which the glutamic acid is substituted with alanine (Figure 3A-B). Indeed, both wild-type hADI1 strains almost completely rescued the fecundity defect in the *Dadi1* mutants (Figure 3C). In contrast, hADI1-E94A could not rescue the fecundity defect in *Dadi1* mutant females (Figure 3D), despite that the protein levels of E94A mutants and wild-type-15 of hADI1 were equal. Flies expressing this E94A mutant hADI1 displayed similar egg production ability to those expressing the GFP control protein. Based on these data, we concluded that human ADI1 is functionally interchangeable with *Drosophila* ADI1 and that the enzymatic activity of ADI1 is essential for normal egg production.

### Methionine supply suppressed the fecundity defect in *Dadi1* mutant females

**Figure 4:**
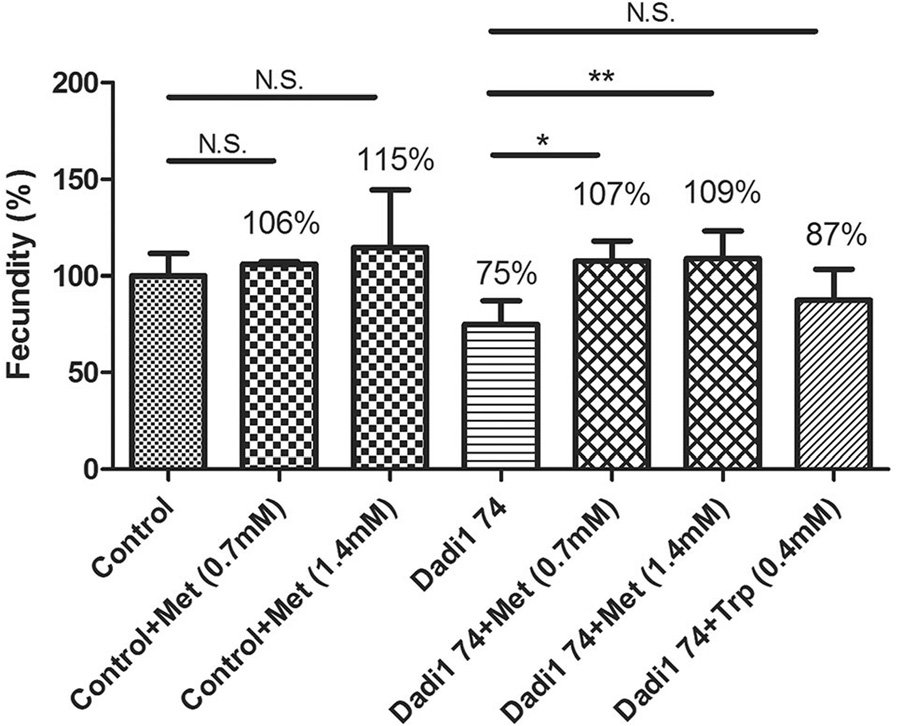
**Methionine supplementation rescued the fecundity defect in*Dadi1*mutant alleles.** The fecundity of *Dadi1* null mutants was recovered to the level of control flies after adding back met but not trp. *P < 0.05, **P < 0.01 and N.S., no significance.

The MTA cycle regulates the methionine metabolism. Thus we set out to test whether providing a supply of methinonine could rescue the fecundity defect in *Dadi1* mutants. Indeed, adding 0.7 mM or 1.4 mM methionine to the fly food completely rescued the fecundity defect (Figure 4), such that the mutant females showed a similar ability to produce eggs to that of the control females. To further investigate whether the rescue was specifically due to methionine, tryptophan was also supplied in the food. However, no significant rescue in fecundity was found as a result of supplying tryptophan (Figure 4). Therefore, we concluded that the depletion of methionine was the major cause for reduced fecundity in *Dadi1* mutants.

### Metabolites of MTA cycle were altered in *Dadi1* mutant ovaries

**Figure 5:**
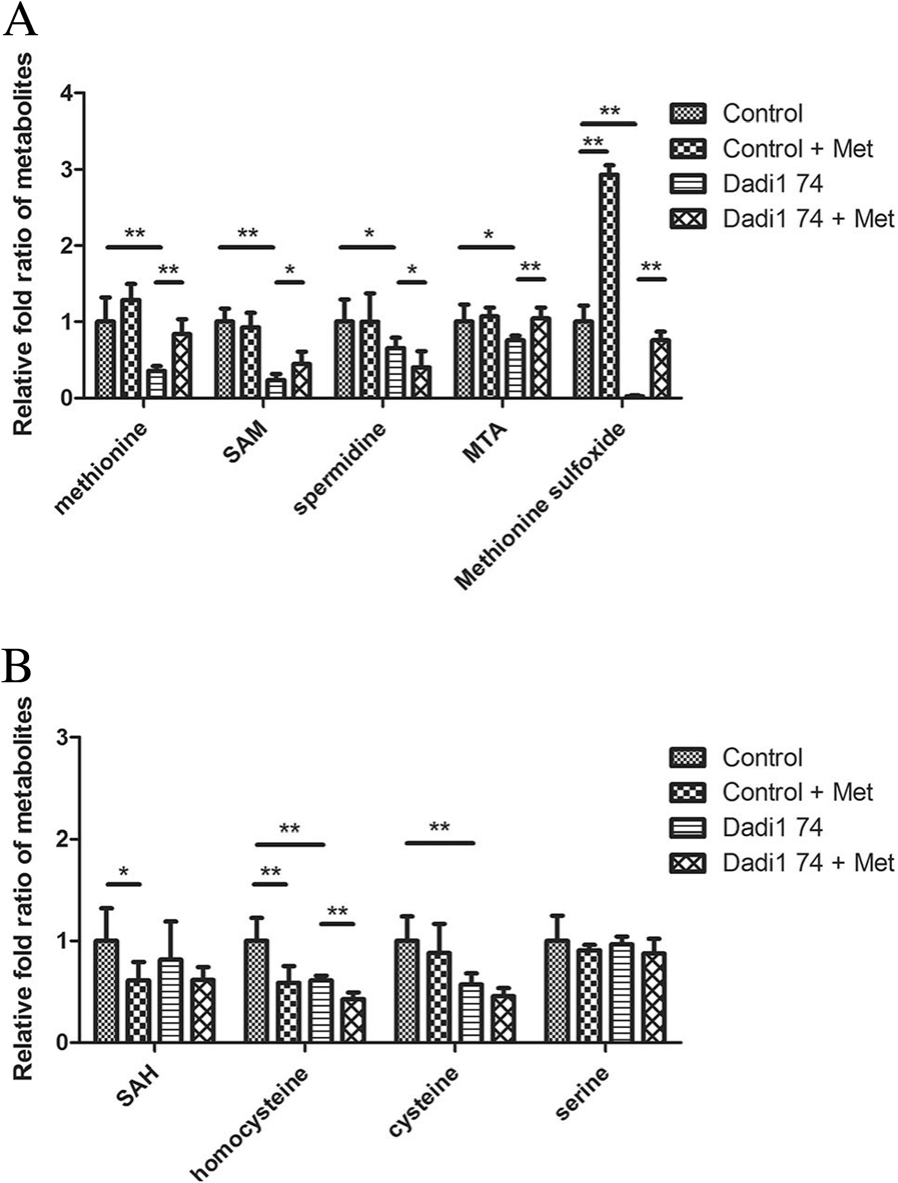
**The metabolites of MTA cycle are affected by*D*ADI1. (A)** Four metabolites, Methionine, SAM, MTA and Methionine sulfoxide, were observed to be rescued in *Dadi1* mutant ovaries by methionine addition. **(B)** Under the restricted diet condition, the metabolites of the methyl cycle and MTA cycle were dramatically changed in *Dadi1* mutant ovaries. The raw data of metabolites are displayed below the picture. The results represent the means ± SD of three experiments. *P < 0.05, **P < 0.01 and N.S. indicates no significance.

In order to understand what metabolites are critical for fly fecundity, we first examined the metabolism in *Dadi1* mutant ovaries and in the ovaries from control flies in dietary restriction condition. UPLC-TQMS based targeted metabolites analysis indicated that metabolites in the MTA cycle, including methionine, SAM, MTA, and spermidine were significantly reduced. It clearly displayed that MTA cycle are significantly impaired in *Dadi1* mutant ovaries (Figure 5A). Consistently, the metabolites in the downstream of ADI1 (methionine and SAM) were affected more severely than those upstream (MTA and spermidine). However, the metabolites in methyl cycle (SAH and homocysteine) and the trans-sulfuration (cysteine) were also affected, suggesting that under dietary restriction condition the methionine metabolism was greatly reduced in the *Dadi1* mutant ovary (Figure 5B). The contents of Methionine sulfoxide, an oxidized form of methionine, were dramatically reduced. In contract, the levels of serine and phenylalanine, an indication of amino acid pools, remained unaffected (Figure 5B), suggesting that the general amino acid metabolism was not altered.

Since methionine supply in the diet rescued fecundity defect in *Dadi1* mutant completely (Figure 4), we further compared the methionine metabolism in *Dadi1* mutant ovaries and mutant ovaries with a methionine supply in the diet. Results demonstrated that the methionine supply elevated metabolites of MTA cycle, such as methionine, SAM, and MTA. Interestingly, the contents of spermidine were not increased. Nevertheless, metabolites in the methyl cycle and trans-sulfuration were not increased. These results suggest that MTA cycle might be dominant in methionine metabolism in fly ovary under dietary restriction condition. The amount of Methionine sulfoxide was also returned to the levels of control ovaries (Figure 5B). Again, the contents of serine and phenylalanine remain the same, indicating this amount of methionine in food supply did not alter general amino acid polls. Collectively, these results suggest that the rescue of fecundity by methionine most likely relates to the metabolites of the MTA cycle.

## Discussion

### *Drosophila* ADI1 is involved in MTA cycle

In the present study, we demonstrated that the MTA cycle is crucial for *Drosophila* female fecundity under the restricted diet condition, and that a methionine supply could suppress the fecundity defect in *Dadi1* mutants. The role of MTA cycle is used for the SAM, the principal methyl donor, which can recycle sulfur group to regenerate methionine. The MTA cycle also links tightly with polyamine synthesis. The intermediate compound of MTA cycle, MTA, is formed from dcSAM and a polyamine precursor [[15]]. In addition, the downstream products of MTA cycle, MTA and MTOB, are reported to inhibit the rate-limiting enzyme in polyamine synthesis, ornithine decarboxylase [[28]]. However, the biological functions of MTA cycle are still poorly understood. In previous studies, the MTA cycle has been found to process 10 ~ 15% of methionine contents in yeast [[29]], and is active in the human liver and kidney. Similarly, the MTA cycle is very active in *Drosophila* ovaries, which was revealed by the observation of a 64% reduction of methionine which could not be recycled back through the MTA cycle in *Dadi1* mutant ovaries (Table 1).

**Table 1:** The levels of biosynthetic metabolites

				**(ppm)**
**Metabolites**	**Control**	**Control + Met**	**Dadi1-74**	**Dadi1-74 + Met**
SAM	6.733 ± 1.149	6.217 ± 1.295	1.604 ± 0.535	3.014 ± 1.061
Spermidine	0.647 ± 0.188	0.647 ± 0.241	0.422 ± 0.087	0.262 ± 0.134
MTA	0.013 ± 0.003	0.014 ± 0.002	0.010 ± 0.001	0.014 ± 0.002
Methionine sulfoxide	15.852 ± 3.377	46.482 ± 1.972	0.392 ± 0.206	11.949 ± 1.794
SAH	0.028 ± 0.009	0.017 ± 0.005	0.023 ± 0.011	0.017 ± 0.004
Homocysteine	0.083 ± 0.019	0.049 ± 0.014	0.051 ± 0.004	0.035 ± 0.005
Cysteine	0.261 ± 0.063	0.230 ± 0.075	0.149 ± 0.029	0.119 ± 0.021
Serine	11.839 ± 2.942	10.746 ± 0.643	11.450 ± 0.894	10.374 ± 1.722

The concentrations of metabolites were analyzed by UPLC-MS/MS and normalized with phenylalanine.

The unit of concentration is ppm (parts per million).

### *Drosophila* fecundity is regulated by *D*ADI1 through the alteration of methionine balance

*Drosophila* female fecundity is controlled by nutrients intake and signaling pathways. For example, flies fed with restriction food can extend adult survivorship but reduce fecundity [[30],[31]]. Moreover, scientists report that mutants in the insulin/insulin-like growth factors (IGFs) signaling pathway have reduced juvenile hormone contents, prolonged lifespan and impaired in reproduction [[32]-[34]]. Protein supply is considered very crucial for the growth and reproduction of organisms [[11]]. Recently, among all essential amino acids, methionine has been found to play the most pivotal role in *Drosophila* fecundity. Partridge et al. showed that adding methionine alone in restricted diet conditions food promoted longevity and increased fecundity [[11]]. When the maternal diets with an inappropriate intake of methionine can affect short-term reproductive ability and impair long-term health of the offspring [[35]]. A recent study suggested that the rat diet with low protein during gestation also observes the alterations of DNA methylation and gene expression in the offspring [[36]]. Furthermore, several studies also demonstrated that methionine is able to regulate gene expression and protein synthesis through the target of the rapamycin (mTOR) pathway [[37],[38]]. The mTOR activity is modulated by the content of amino acids and insulin/insulin-like growth factors (IGFs). In contrast, adding methionine did not promote egg production in mutant flies that express a dominant- negative form of insulin receptor [[11]]. So, we propose that the MTA cycle may control methionine balance in *Drosophila* and lead to normal egg laying through the regulation of amino acid signals (via the mTOR pathway and insulin pathway). Further experiments are needed to reveal the details of how any mechanism controlled by methionine affects fecundity.

### The metabolites of methionine metabolism are affected by *D*ADI1

Given that an increased methionine supply did not improve fecundity much in the control flies (Figure 4A), even as it increased the level of Methionine sulfoxide dramatically (by about 2 folds), we concluded that Methionine sulfoxide may not be the critical metabolite regulating fecundity. However, it is still possible that Methionine sulfoxide plays a role at the check-point of oogenesis progression. Therefore, when the content is below some threshold, the egg production is limited. This could explain why Methionine sulfoxide did not show a dosage effect on fecundity. In addition, spermidine, which is known to play a major role in cell proliferation, was not increased much with methionine supply (Figure 4B). Therefore, we hypothesized that the role of the MTA cycle in fly fecundity may not simply be to affect cell proliferation through control of spermidine contents. On the other hand, the content of spermidine in the fly ovary is much more than that required for normal fecundity, even under a restricted diet condition or in *Dadi1* mutants. Based on the almost 100% recovery in metabolite contents, MTA may correlate with the rescued fecundity. MTA is known as a methyltransferase inhibitor, which influence methylation status [[39]]. Because the contents of MTA are very low in fly ovary, further experiments are needed to confirm the importance of MTA in fly fecundity. SAM is a universal methyl group donor for DNA, RNA, protein and lipid. In the fly ovary, SAM-S seems to be very active, leading to rapid conversion of methionine into SAM, such that SAM levels are 10 times greater than those of methionine. However, which of these three metabolites plays the most critical role and how it regulates fecundity are questions that remain to be resolved by further experiments.

### The relationship of reproduction and longevity is uncoupled in *Dadi1* mutant allele

Reduced fecundity has been found to be coupled with extended life spans in many organisms [[26],[40]]. In *Drosophila*, protein supply played a pivotal role in female fecundity, and under the restricted diet condition fewer eggs were produced and the average life span was prolonged. Interestingly, adding back essential amino acids resulted in fecundity being recovered, whereas life span was reduced. However, supplying methionine appears to uncouple fecundity and life span, such that females lay normal numbers of eggs and while living just as long as control flies [[11]]. In *Dadi1* mutants, the methionine content in ovaries was reduced to 36% of that in control flies, and the fecundity was diminished by about 20-30% (Figures 2 and 3 and Table 1). However, *Dadi1* mutants were less viable (5% lethality) and had a shorter average life span (data not shown). It is possible that this reduced life span was caused by a metabolic imbalance. Consistently, the methionine supply did not alter the life span of *Dadi1* mutants.

## Conclusions

In summary, this study presents the first isolation of MTA cycle enzyme mutant alleles in *Drosophila*. The MTA cycle enzyme, *D*ADI1, is required for normal fecundity. The fact that human ADI1 can rescue the egg production defect suggests that the functions of ADI1 proteins among different species are interchangeable and that the enzymatic activity of ADI1 is essential for its ability to regulate fly reproductive activity. The fecundity defect in *Dadi1* mutants was rescued by adding methionine under dietary restriction. Using metabolic analysis, we found that *D*ADI1 may regulate fly fecundity through the change of MTA cycle metabolites. The discovery of the *D*rosophila ADI1 protein in this study could clarify the role of the MTA cycle in methionine metabolism. Furthermore, the results of this study suggest that normal fecundity may be controlled by the metabolites of methionine metabolism.

## Competing interest

The authors declare that they have no competing interests.

## Authors’ contributions

Conceived and designed the experiments: HYC, LMP and YHL; Performed experiments: HYC, YHL, GLS and HYT; Data analysis: HYC, YHL, MLC and MSS; Manuscript writing: HYC and LMP. All authors have read and approved the manuscript.
